# Automatic inference of ICD-10 codes from German ophthalmologic physicians’ letters using natural language processing

**DOI:** 10.1038/s41598-024-59926-3

**Published:** 2024-04-19

**Authors:** D. Böhringer, P. Angelova, L. Fuhrmann, J. Zimmermann, M. Schargus, N. Eter, T. Reinhard

**Affiliations:** 1 Eye Center of the University Hospital Freiburg, Medical Faculty of the Albert-Ludwigs-University Freiburg, Freiburg, Germany; 2 Department of Ophthalmology, Asklepios Hospital Nord-Heidberg, Hamburg, Germany; 3 Department of Ophthalmology, Medical Center, University of Münster, Münster, Germany

**Keywords:** Diagnosis coding, Artificial intelligence, Natural language processing, Clinical registries, Data mining, Epidemiology

## Abstract

Physicians’ letters are the optimal source of diagnoses for registries. However, most registries demand for diagnosis codes such as ICD-10. We herein describe an algorithm that infers ICD-10 codes from German ophthalmologic physicians’ letters. We assess the method in three German eye hospitals. Our algorithm is based on the nearest-neighbor method as well as on a large thesaurus for ICD-10 codes. This thesaurus was embedded into a Word2Vec space created from anonymized physicians’ reports of the first hospital. For evaluation, each of the three hospitals sent all diagnoses taken from 100 letters. The inferred ICD-10 codes were evaluated for correctness by the senders. A total of 3332 natural language terms had been sent in (812 hospital one, 1473 hospital two, 1047 hospital three). A total of 526 non-diagnoses were excluded upfront. 2806 ICD-10 codes were inferred (771 hospital one, 1226 hospital two, 809 hospital three). In the first hospital, 98% were fully correct and 99% correct at the level of the superordinate disease concept. The percentages in hospital two were 69% and 86%. The respective numbers for hospital three were 69% and 91%. Our simple method is capable of inferring ICD-10 codes for German natural language diagnoses, especially when the embedding space has been built with physicians’ letters from the same hospital. The method may yield sufficient accuracy for many tasks in the multi-centric setting and can easily be adapted to other languages/specialities.

## Introduction

Secondary use of electronic healthcare records for registry research requires the consistent and comprehensive extraction of diagnoses. Reimbursement diagnoses are an obvious choice because these already come in a code system such as the International Classification of Diseases 10 (ICD-10)^1^. However, administrative diagnoses are potentially incomplete because the medical history is commonly omitted^2,3^. Consequently, additional clinical data improved performance in comparison to using reimbursement diagnoses alone in a specific classification task^4^.

The physicians’ reports are a better source for diagnoses because they usually cover the medical context more comprehensively in comparison to reimbursement diagnoses. The major downside is that natural language needs the transformation into a code system before the data can be used in registries^5^.

This transformation is complicated by the abundance of grammatical variants in physicians’ reports. These necessitate sophisticated grammatical algorithms specifically adopted for the medical domain^6–8^. Such methods have been recently proposed for English medical texts but not for medical texts in German yet. Consequently, we are not aware of a method to automatically infer ICD-10 codes from German ophthalmological physicians’ reports in natural language.

Word vector embeddings are a language-neutral and grammar-free purely data driven approach and have already been used in many medical natural language processing tasks and applications^9^. Word vector embeddings map words into a multidimensional number space on the basis of semantic relatedness alone. Word2vec e.g. uses a shallow neural network for this purpose^10^. Word2vec is a method for creating word embeddings, which are vector representations of words capturing their semantic relationships.

We herein describe a methodology to infer ICD-10 codes from ophthalmological physicians’ notes in German language based on Word2vec embeddings created from two gigabytes worth of historical physicians’ reports from one university eye hospital in combination with a large thesaurus of ICD-10 codes.

We evaluate the performance of our method in this hospital and additionally in two other German eye hospitals. This was done to assess how far the method is suitable to the multi-centric setting, such as for automated feeding into a nation-wide ophthalmologic registry without specific adaptations to each of the contributors.

## Methods

### Building the embedding space

Our embedding is based on the Word2vec method, which maps words into a multidimensional number space based on semantic relatedness as described previously^5^. We used a ‘phrases-file’ extension^11^ to maintain the meaning of multi-word diagnoses. The phrases file comprised annotations from the ‘Alpha-ID’ catalog and all annotations to ICD-10 codes entered into the administrative database of the University Hospital Freiburg over the last 15 years. The corpus file contained plain text from anonymized physicians’ reports written between 2003 and 2018 at the Eye Center of the University Hospital Freiburg. Pre-processing steps included eliminating numbers higher than 10, non-alphanumeric characters, splitting composite words, transcribing German umlauts, and converting all words to lowercase. The Word2vec executable was run with specific settings, such as a window size of 5 words and 5 iterations to optimize the neural network. For example, For example, the word ‘cataract’ might be represented by a 300-dimensional vector [0.12, − 0.08, …, 0.23], capturing its semantic context within the corpus. A multi-word phrase such 'rhegmatogenous retinal detachment' would be treated as a single entity in the embedding space, preserving its semantic meaning. The resulting embedding space comprises a vocabulary of 347,370 distinct words or phrases in 300 dimensions.

### Annotating the embedding space

We compiled a comprehensive ICD-10 thesaurus from the aforementioned Alpha-ID catalog together with the manual annotations to ICD-10 codes that had been entered into the administrative database of the University Hospital Freiburg. After cleanup and removing duplicates we obtained a coding thesaurus with 84,419 natural language annotations for 16,132 distinct ICD-10 codes. We annotated the embedding space with this thesaurus by means of ‘placing’ the centroids of all embedded thesaurus entries for each ICD-10 code. These centroids serve as the targets for the nearest neighbor search as described below.

### Inference of ICD-10 codes

We opted for nearest-neighbor search to link inference queries to the most appropriate ICD-10 code. When a query comprises multiple words, we numerically average the embedding vectors column-wise. When the query comprises a phrase from the phrases file, the phrase embedding is concatenated to the vector array before averaging. Finally we look for the nearest ICD-10 cluster in embedding space to the averaged embedding of the query sequence. We also calculate the cosine distance between the query embedding and the centroids of the ICD-10 cluster. This enables thresholding to reduce misclassifications in case no sensible neighbor is found. Model inference was implemented using the R system. We set up an intranet web service for our inference studies.

### Validation study

We included a total of three German eye hospitals with no overlap in personnel or patients. The Eye Center of the University Hospital Freiburg, the source of the Word2vec embedding space, was the first hospital (H1). Here, we made sure that none of the physicians’ reports of the evaluation study had been part of the corpus that was used for generating the embeddings. The second hospital (H2) was the Department of Ophthalmology, Asklepios Hospital Nord-Heidberg, Hamburg, Germany. The third hospital (H3) comprised the Department of Ophthalmology, University of Münster Medical Center.

We requested all diagnosis segments of 100 physicians’ reports from all three hospitals. All data were manually anonymized at the sending hospital in order to ensure complete anonymity before sending the texts away for inference. The diagnosis segments were extracted from the reports using regular expressions in the perl programming language. All segments were manually reviewed to remove non-diagnoses such as medication mentions upfront. Inference was performed using an intranet based implementation of our algorithm. The inferred ICD-10 codes were returned to the senders for evaluation. Evaluation comprised assessment whether the ICD-10 code was either completely correct or, in a secondary assessment, at least correct for the superordinate disease group (e.g. rhegmatogenous retinal detachment and serous retinal detachment both are retinal detachments).

### Ethics and data protection

We did not need to seek advice from the ethics committees because the data of this study had been anonymous from the start. It was neither possible nor necessary to obtain informed consent from the patients for the same reason. All methods were carried out in accordance with relevant guidelines and regulations.

### Ethical approval

Ethics approval was not needed because all data in this study were anonymous from the start. In Germany, research with data that cannot be traced back to natural persons is not regulated by the professional codes of the medical associations. We therefore did not seek ethical advice as we only used anonymized data from the start. Furthermore, the EU GDPR does not apply to anonymized data (detailed in recital 26 of the EU GDPR). On this basis, we herewith confirm that all methods were carried out in accordance with relevant guidelines and regulations.

## Results

A total of 3332 word sequences (812 H1, 1473 H2, 1047 H3) were extracted. 2806 were identified as diagnoses. A total of 2806 ICD-10 codes were inferred by our methodology (771 H1, 1226 H2, 809 H3). In H1, 98% were classified as completely correct (CC) and 99% as correct (C) concerning the superordinate disease concept. The percentages in H2 were 69% (CC) and 86% (C). The respective numbers for H3 were 69% (CC) and 91% (C). Examples for correctly and incorrectly inferred ICD-10 codes are shown alongside the input texts in Table 1.

**Table 1:** Representative examples from the validation study.

Input phrase (German)	English translation	Inferred ICD-10 code	ICD-text	Considered completely correct (CC)	Considered correct at the level of the superordinate concept (C)
Sekundärglaukom	Secondary glaucoma	H40.5	Glaucoma (secondary) following other affections of the eye	Yes	Yes
bei Z.n. Zentralvenenverschluss	following central venous occlusion	H34.8Z	Retinal vein occlusion	Yes	Yes
Anamnestisch Unverträglichkeiten gegen Glaukomtropfen	History of intolerances to glaucomatous eye drops	Z88.2	Self reported allergy against sulfonamides	No	Yes
AMT Overlay	AMT overlay	T83.4	Mechanical complication from other protheses, implants or grafts in the genital tract	No	No

Table 2 presents the CC-accuracy of ICD-10 code inference for different disease groups across the three hospitals. Common eye disorders such as disorders of refraction and accommodation (H52), senile cataract (H25), and disorders of the vitreous body (H43) achieved high accuracy rates (> 80%) across all hospitals. However, the accuracy for some disease groups, such as other retinal disorders (H35) and disorders of the globe (H44), varied considerably between hospitals. Rare diseases, denoted by an asterisk (*), generally had lower accuracy rates, with some conditions like congenital malformations of the posterior segment of the eye (Q14) and failure and rejection of transplanted organs and tissues (T86) having 0% accuracy in H3.

**Table 2:** Percentage CC-Accuracy of ICD-10 code inference for different disease groups across the three hospitals.

ICD 10 group (*rare diseases)	H1	H3	H2
H52 Disorders of refraction and accommodation	100.00	100.00	93.10
H25 Senile cataract	100.00	100.00	87.50
H43 Disorders of vitreous body	100.00	100.00	80.00
H47 Disorders of optic [2nd] nerve and visual pathways	100.00	100.00	75.00
I10 Essential (primary) hypertension	100.00	98.04	81.25
Z96 Presence of other functional implants	100.00	93.07	93.62
H40 Glaucoma	100.00	90.91	56.82
H34 Retinal vascular occlusions	100.00	88.89	87.50
Z98 Other postprocedural states	100.00	85.96	93.81
H53 Visual disturbances	100.00	85.71	66.67
H04 Disorders of lacrimal system	100.00	80.00	88.89
Z95 Presence of cardiac and vascular implants and grafts	100.00	62.50	66.67
H02 Other disorders of eyelid	100.00	60.00	90.00
H33 Retinal detachments and breaks	100.00	59.46	31.82
H26 Other cataract	100.00	55.56	–
H18 Other disorders of cornea	100.00	53.85	71.43
Z92 Personalhistory of medical treatment	100.00	45.45	78.95
E11 Type 2 diabetes mellitus	100.00	11.76	7.69
H16 Keratitis	100.00	–	81.25
D90 Immune mechanism disorders, not elsewhere classified*	100.00	–	57.14
C44 Other and unspecified malignant neoplasm of skin*	100.00	–	–
H01 Other inflammation of eyelid	100.00	–	–
H10 Conjunctivitis	100.00	–	–
H17 Corneal scars and opacities	100.00	–	–
H36 Retinal disorders in diseases classified elsewhere*	100.00	–	–
L23 Allergic contact dermatitis	100.00	–	–
S05 Injury of eye and orbit	100.00	–	–
S06 Intracranial injury	100.00	–	–
H35 Other retinal disorders	98.08	91.21	66.67
Z51 Other medical care	85.71	–	80.00
E01 Iodine-deficiency-related thyroid disorders and allied conditions*	–	100.00	–
E78 Disorders of lipoprotein metabolism and other lipidemias	–	100.00	–
H54 Visual impairment including blindness (binocular or monocular)	–	100.00	–
T78 Adverse effects, not elsewhere classified	–	100.00	–
H50 Other strabismus	–	87.50	60.00
I25 Chronic ischaemic heart disease	–	75.00	–
H44 Disorders of globe	–	62.50	80.00
Z88 Personal history of allergy to drugs, medicaments and biological substances	–	50.00	–
Q14 Congenital malformations of posterior segment of eye*	–	–	00.00
T86 Failure and rejection of transplanted organs and tissues*	–	–	00.00
Z01 Other special examinations and investigations of persons without complaint or reported diagnosis	–	–	88.89
Z94 Transplanted organ and tissue status	–	–	81.82
Z83 Family history of other specific disorders*	–	–	80.00
H11 Other disorders of conjunctiva	–	–	60.00
H20 Iridocyclitis	–	–	40.00

Rare diseases are denoted by an asterisk (*). ICD codes occurring only three times or less in the dataset were excluded upfront.

Notably, H1 achieved 100% CC accuracy for most disease groups, likely due to its contribution to the training data. H2 and H3 showed lower accuracy rates for certain disease groups, such as type 2 diabetes mellitus (E11) and other retinal disorders (H35). Some disease groups, such as iodine-deficiency-related thyroid disorders (E01) and personal history of allergy to drugs, medicaments, and biological substances (Z88), were only present in H3, while others, such as transplanted organ and tissue status (Z94) and family history of other specific disorders (Z83), were only found in H2.

## Discussion

Our data indicate that it is possible to infer ICD-10 codes from routine German physicians’ reports at high precision. This especially holds when the training data closely match the queries as had been the case in the first hospital. Although we ensured that there was no direct overlap between the letters from the training corpus and the validation study, some physicians may have contributed text material to both the training corpus and also to the validation study. We speculate that characteristic and stable language preferences may have improved the performance in the validation study of the first hospital, e.g. by means of reducing out-of-vocabulary queries. Another reason for the excellent performance in the first hospital is that the ICD thesaurus had been derived from all manual annotations to ICD-10 encodings retrieved from the administrative database of the same hospital. This ensured that all non-ophthalmological diagnoses of all specialities in this large university hospital are covered, including rare disease peculiarities. Both advantages had not been available at the other two hospitals.

A use case of our method is to provide diagnoses for the nation-wide registry “oregis”, a subsidiary of the German Ophthalmic Society, directly from physicians’ reports inside a special adaptor software installed locally^12^. This approach would be feasible only if the inference also worked for hospitals or clinics that did not contribute to the training corpus and thesaurus. This is why we included the two other hospitals into our validation study. It is important to note that the second hospital was not a university center but a regional hospital with a patient spectrum more similar to office-based ophthalmologists in comparison to the university hospitals.

Interestingly, H2 and H3 yielded relatively high levels of correctness, which may be sufficient for downstream tasks focusing on more common diseases and relying on relative comparisons, such as temporal changes or cross-sectional analyses, rather than absolute numbers.

However, other use cases may demand the higher degrees of accuracy observed in H1. Out-of-vocabulary queries were identified as major contributors to the performance degradation in H2 and H3. These queries were primarily attributed to center-specific abbreviations or diagnosis synonyms. For example, H2 frequently used the abbreviation 'PDRP' for proliferative diabetic retinopathy, which is not commonly used in H1. Additionally, non-ophthalmological and rare disease diagnoses arising from local peculiarities may also contribute to the performance differences. For instance, H3 utilized abbreviations such as "KDIGO," suggesting a greater involvement in treating patients with kidney disorders compared to H1.

Such local peculiarities could be a source of bias in the unmodified application of our method when feeding "oregis". Specific diagnoses may be systematically under-reported due to hospital-specific variations in terminology and falsely pretend regional variations in prevalence or incidence of certain diseases. Given the inclusiveness of oregis with the aim to eventually reach all German ophthalmologists, the method should be refined to become more robust against regional variations in the physicians’ letters.

Because the computational resources needed by our method are rather limited, the Word2vec embedding could be easily redone overnight after extending the training corpus with historical physicians’ letters from other sites. This could even be done progressively just before new sites are added. These improvements would most likely mitigate the degradation of inference accuracy from novel sites and bring the overall performance closer to the one from the first hospital in our validation study.

A major challenge for disseminating this method to other hospitals or medical-surgical disciplines, however, will be to guarantee compliance with the General Data Protection Regulation of the European Union. Automated anonymisation systems have already been proposed for English datasets^13^. However, we are not aware of de-identification tools for the German language that are sufficiently robust for giving away large numbers of physicians’ letters to a third party without consent from the patients. For this reason, our method has currently to be locally implemented at each site separately. Obviously, more research is warranted in this field in order to enable a continuously growing corpus of clinical text for making this available 'of-the-shelf' for the broader medical domain in Germany.

## Conclusion

In conclusion, our study demonstrates the feasibility of automatically inferring ICD-10 codes from German ophthalmology medical records using a Word2vec-based approach. The proposed method achieved high accuracy, particularly when trained on data from the same hospital. However, performance variations across hospitals highlight the need for further refinement and adaptation to hospital-specific terminologies. Future work should focus on expanding the training corpus, improving the handling of rare diseases, and facilitate the application's dissemination to other medical domains and institutions.

## Data Availability

The data from this manuscript are not publicly available due to privacy concerns from the healthcare providers, despite anonymization. Individual access to the data can be arranged, given appropriate precaution measures. Please send requests via email to: daniel.boehringer@uniklinik-freiburg.de.

## Code availability

The code is available here: https://github.com/daboe01/InferenceOfICD10CodesFromText.

## References

[1] Ewing C (2016). Unlocking the benefits of ICD-10 through data analytics. J. AHIMA.

[2] Owodunni OP (2021). Systematic undercoding of diagnostic procedures in National Inpatient Sample (NIS): A threat to validity due to surveillance bias. Qual. Manag. Health Care.

[3] Jackson ML, Nelson JC, Jackson LA (2011). Why do covariates defined by international classification of diseases codes fail to remove confounding in pharmacoepidemiologic studies among seniors?. Pharmacoepidemiol. Drug Saf..

[4] Liao KP (2010). Electronic medical records for discovery research in rheumatoid arthritis. Arthritis Care Res..

[5] Böhringer D, Lang SJ, Daniel MC, Lapp T, Reinhard T (2019). Unsupervised linkage between ICD- and alpha-ID codes and real-world diagnoses from medical reports by means of the ‘word2vec’ method. Klin. Monbl. Augenheilkd..

[6] Dietrich G (2018). Ad Hoc information extraction for clinical data warehouses. Methods Inf. Med..

[7] Jiang M (2015). Parsing clinical text: How good are the state-of-the-art parsers?. BMC Med. Inform. Decis. Mak..

[8] Zhang Y, Zhang Y, Qi P, Manning CD, Langlotz CP (2021). Biomedical and clinical English model packages for the Stanza Python NLP library. J. Am. Med. Inform. Assoc..

[9] Wu S (2020). Deep learning in clinical natural language processing: A methodical review. J. Am. Med. Inform. Assoc..

[10] Mikolov, T., Chen, K., Corrado, G. & Dean, J. Efficient estimation of word representations in vector space. 10.48550/ARXIV.1301.3781 (2013).

[11] Artetxe, M., Labaka, G. & Agirre, E. A robust self-learning method for fully unsupervised cross-lingual mappings of word embeddings. In *Proceedings of the 56th Annual Meeting of the Association for Computational Linguistics (Volume 1: Long Papers)* (Association for Computational Linguistics, 2018). 10.18653/v1/p18-1073

[12] oregis—Deutsches Ophthalmologisches Register. https://oregis.de/.

[13] Neamatullah I (2008). Automated de-identification of free-text medical records. BMC Med. Inform. Decis. Mak..

